# The Association Between Naples Prognostic Score and Coronary Collateral Circulation in Patients with Chronic Coronary Total Occlusion

**DOI:** 10.3390/diagnostics15192500

**Published:** 2025-10-01

**Authors:** Abdullah Tunçez, Sevil Bütün, Kadri Murat Gürses, Hüseyin Tezcan, Aslıhan Merve Toprak Su, Burak Erdoğan, Mustafa Kırmızıgül, Muhammed Ulvi Yalçın, Yasin Özen, Kenan Demir, Nazif Aygül, Bülent Behlül Altunkeser

**Affiliations:** Department of Cardiology, Faculty of Medicine, Selçuk University, Konya 42130, Türkiye; svl.btn@gmail.com (S.B.); kmuratg@yahoo.com (K.M.G.); drhuseyintezcan@hotmail.com (H.T.); aslihantoprak93@gmail.com (A.M.T.S.); brkrdgn0@gmail.com (B.E.); mkgul96@gmail.com (M.K.); ulviyalcin@gmail.com (M.U.Y.); ysnozn70@gmail.com (Y.Ö.); drkenan76@yahoo.com (K.D.); nazifaygul@gmail.com (N.A.); bbaltunkeser@hotmail.com (B.B.A.)

**Keywords:** chronic total occlusion, coronary collateral circulation, Naples prognostic score, high-density lipoprotein cholesterol, rentrop classification

## Abstract

**Background:** Coronary collateral circulation (CCC) plays a crucial protective role in patients with chronic total occlusion (CTO), mitigating ischemia and improving long-term outcomes. However, the degree of collateral vessel development varies substantially among individuals. Systemic inflammatory and nutritional status may influence this variability. The Naples Prognostic Score (NPS) is a composite index reflecting these parameters, yet its relationship with CCC remains incompletely defined. **Methods:** We retrospectively analyzed 324 patients with angiographically confirmed CTO at Selçuk University Faculty of Medicine between 2014 and 2025. Coronary collaterals were graded using the Rentrop classification, and patients were categorized as having poor (grades 0–1) or good (grades 2–3) collaterals. The NPS was calculated using serum albumin, cholesterol, neutrophil-to-lymphocyte ratio, and lymphocyte-to-monocyte ratio. Baseline clinical and laboratory data were compared between groups. Univariate and multiple binary logistic regression analyses were performed to identify independent predictors of collateral development. **Results:** Of the 324 patients, 208 (64.2%) had poor and 116 (35.8%) had good collateral circulation. Patients with good collaterals had higher body mass index, HDL Cholesterol (HDL-C), and triglyceride levels, and significantly lower NPS values compared with those with poor collaterals (*p* < 0.05 for all). In multiple binary logistic regression analysis, HDL-C (OR 1.035; 95% CI 1.008–1.063; *p* = 0.011) and NPS (OR 0.226; 95% CI 0.130–0.393; *p* < 0.001) emerged as independent predictors of well-developed collaterals. **Conclusions:** Both NPS and HDL-C are independently associated with the degree of coronary collateral circulation in CTO patients. These findings highlight the interplay between systemic inflammation, nutritional status, lipid metabolism, and vascular adaptation. As simple and routinely available measures, NPS and HDL-C may serve as practical tools for risk stratification and identifying patients at risk of inadequate collateral formation. Prospective studies with functional assessments of collateral flow are warranted to confirm these associations and explore potential therapeutic interventions.

## 1. Introduction

Chronic coronary total occlusion (CTO) is a complex and clinically significant manifestation of coronary artery disease (CAD), defined as the complete occlusion of a coronary artery for at least three months with thrombolysis in myocardial infarction (TIMI) grade 0 flow [1]. CTOs are present in approximately 15–20% of patients undergoing coronary angiography and are associated with increased morbidity and mortality, including higher risks of heart failure, ventricular arrhythmias, and adverse cardiovascular events [2,3]. Despite advances in percutaneous coronary intervention (PCI), the management of CTO remains challenging, with success rates highly dependent on anatomical complexity and operator expertise [4].

In patients with CTO, the development of coronary collateral circulation (CCC) serves as a critical compensatory mechanism, providing alternative blood flow to ischemic myocardium and mitigating the detrimental effects of persistent occlusion [5]. Well-developed collaterals have been associated with improved myocardial viability, reduced infarct size, and better long-term clinical outcomes [6,7]. However, collateral formation varies significantly among individuals, with some patients exhibiting robust collateral networks while others show minimal adaptive response [7,8]. This heterogeneity suggests that systemic factors—such as inflammation, endothelial dysfunction, and metabolic derangements—play a crucial role in modulating collateral vessel development [9,10].

The Naples Prognostic Score (NPS) is an integrative biomarker-based scoring system initially developed to assess the inflammatory and nutritional status of patients with cancer, but it has since demonstrated prognostic value in cardiovascular diseases [11,12,13]. The NPS incorporates serum albumin, total cholesterol (TC), the lymphocyte-to-monocyte ratio (LMR), and the neutrophil-to-lymphocyte ratio (NLR), reflecting a composite of malnutrition and systemic inflammation—two key pathophysiological processes implicated in impaired vascular repair and angiogenesis and first described by Galizia et al. [11] Elevated NLR and hypoalbuminemia, in particular, have been independently linked to endothelial dysfunction and poor collateralization in CAD patients [14,15,16]. Given that chronic inflammation and malnutrition are hallmarks of advanced atherosclerosis, the NPS may serve as a novel tool for risk stratification in CTO patients, potentially predicting the extent of coronary collateralization. Although originally proposed in oncology, the NPS has shown prognostic value in contemporary cardiovascular cohorts, including those with ST elevation myocardial infarction (STEMI), those with Non-ST elevation myocardial infarction (NSTEMI), broad CAD populations (long-term all-cause mortality), and patients undergoing CTO-PCI, supporting its applicability to CTO pathophysiology where systemic inflammation and nutrition plausibly modulate arteriogenesis and collateral growth [11,17,18,19].

While previous studies have explored the relationship between individual inflammatory markers (e.g., C-reactive protein, NLR, LMR) and collateral development, the role of a comprehensive score like the NPS remains underexplored in the context of collateral development in patients with CTO [20,21]. Furthermore, current risk assessment models for CTO revascularization often overlook the systemic inflammatory and nutritional status, despite growing evidence of their influence on cardiovascular outcomes.

Despite its potential prognostic relevance, there is a paucity of data on the relationship between the NPS and coronary collateral degree in patients with CTO. Understanding this association may offer new insights into the pathophysiological interplay between systemic inflammation, nutritional status, and collateral vessel development. By elucidating this relationship, our findings may enhance risk stratification, guide therapeutic decision-making, and identify patients who could benefit from targeted anti-inflammatory or nutritional interventions to improve collateral perfusion with an accessible and cost-effective tool.

Therefore, the present study aims to investigate the association between the NPS and the degree of CCC in patients with chronic coronary total occlusion.

## 2. Materials and Methods

### 2.1. Study Design and Setting

In this single center, retrospective, observational study conducted at Selçuk University Faculty of Medicine, Department of Cardiology, a tertiary care reference center serving a large population in the Central Anatolia, Türkiye. The study protocol was approved by the local institutional ethics committee of Selçuk University Faculty of Medicine (approval code: 2025/165, approval date: 11 March 2025) and conducted in accordance with the Declaration of Helsinki. Due to the retrospective nature of the study, the requirement for informed consent was waived.

### 2.2. Study Population

The study included eligible patients who were diagnosed with CAD and coronary CTO and underwent CTO revascularization procedures between January 2014 and June 2025. Because our center is a reference center in its region, most of the patients are referred patients for CTO revascularization procedure. A CTO was defined as a complete obstruction of a coronary artery with Thrombolysis in Myocardial Infarction (TIMI) flow grade 0, with an estimated duration of at least three months based on clinical history, previous angiograms (if available), and the presence of collateral vessels and angiographic features suggestive of chronicity (e.g., bridging collaterals, blunt stump, or absence of a tapered end) [22].

### 2.3. Inclusion Criteria

Patients were eligible for inclusion if they were 18 years of age or older, had at least one CTO lesion at major epicardial coronary arteries (left anterior descending artery (LAD), left circumflex artery (LCA), and right coronary artery (RCA)) documented by coronary angiography, and had complete medical records, including laboratory results necessary to calculate the NPS.

### 2.4. Exclusion Criteria

Patients were excluded if they had acute coronary syndromes at the time of presentation, severe valvular heart disease and mechanical–bioprosthetic heart valves, active systemic infections, severe trauma or surgical operation within 3 months, known autoimmune or chronic inflammatory diseases, hematological disorders that could affect white blood cell counts, malignancies under active treatment and active chemoradiotherapy, severe hepatic dysfunction (defined as transaminase levels > 5 times the upper normal limit), end-stage renal disease (defined as estimated glomerular filtration rate < 30 mL/min/1.73 m^2^), treatment with hormones or immuno-suppressants, or incomplete laboratory or angiographic data. Patients with active systemic infections (defined as fever ≥ 38 °C, leukocytosis > 11,000/µL, or positive microbiological cultures requiring antimicrobial treatment) were excluded. In addition, patients with known autoimmune or chronic inflammatory diseases (e.g., rheumatoid arthritis, systemic lupus erythematosus, vasculitis, inflammatory bowel disease, psoriasis) were excluded to avoid potential confounding effects on inflammatory and nutritional parameters.

### 2.5. Data Collection and Variables

Patient demographic characteristics (age, sex), cardiovascular risk factors (hypertension, diabetes mellitus (DM), hyperlipidemia, smoking status), past medical history (prior myocardial infarction, prior revascularization), and medication use were retrieved from the hospital’s electronic health record system. Baseline laboratory data, including complete blood count, serum albumin, and blood lipid parameters, were collected from fasting blood samples obtained within 24 h prior to CTO revascularization procedure.

### 2.6. Calculation of the Naples Prognostic Score (NPS)

The NPS was calculated for each patient using four parameters:

1. Serum albumin (≥4 g/dL scored as 0; <4 g/dL scored as 1),
2. Total cholesterol (TC) (≥180 mg/dL scored as 0; <180 mg/dL scored as 1),
3. Neutrophil-to-lymphocyte ratio (NLR) (≤2.96 scored as 0; >2.96 scored as 1),
4. Lymphocyte-to-monocyte ratio (LMR) (>4.44 scored as 0; ≤4.44 scored as 1).

Each parameter was assigned a score of 0 or 1, resulting in a total NPS ranging from 0 to 4. Patients were further categorized into subgroups based on their NPS as low (0–2) or high (3–4), as described in previous studies [11].

### 2.7. Assessment of Coronary Collateral Circulation

All coronary angiograms were reviewed by two experienced interventional cardiologists blinded to the clinical and laboratory data. The degree of CCC was graded using the Rentrop classification system:

- Grade 0: No visible collateral vessels,
- Grade 1: Filling of side branches of the artery to be perfused without visualization of the epicardial segment,
- Grade 2: Partial filling of the epicardial segment via collateral channels,
- Grade 3: Complete filling of the epicardial segment of the occluded artery [23].

In case of disagreement between reviewers, a third cardiologist was consulted, and the final grade was determined by consensus. Patients were classified into two groups according to their collateral development: poor CCC group (Rentrop grades 0–1) and good CCC group (Rentrop grades 2–3).

## 3. Statistical Analysis

All statistical analyses were performed using the Statistical Package for the Social Sciences (IBM SPSS Statistics for Windows, version 21.0; IBM Corp., Armonk, NY, USA). Continuous variables with a normal distribution were expressed as mean ± standard deviation, whereas those not conforming to normality were summarized as median values with interquartile ranges (25th–75th percentile). Categorical variables were presented as absolute frequencies and percentages. The Kolmogorov–Smirnov test was employed to evaluate the assumption of normality. Comparisons of continuous variables were undertaken using Student’s *t*-test or the Mann–Whitney *U* test, according to distributional characteristics. Categorical data were compared using the Chi-square test. Binary logistic regression analysis was performed to determine the independent predictors of coronary collateral degree. Multicollinearity among predictor variables was assessed using variance inflation factors (VIF). All VIF values were <2, indicating no significant collinearity. Variables with a *p*-value < 0.20 in baseline comparisons were entered into univariate logistic regression analyses. Subsequently, only predictors that remained statistically significant (*p* < 0.05) in univariate analyses were included in the multivariate logistic regression model. This stepwise approach was chosen to ensure transparency in variable selection while reducing the risk of model overfitting. Receiver operating characteristic (ROC) curve analysis was applied to assess the discriminative ability of the NPS in predicting poor collateral circulation. The area under the curve (AUC) with 95% confidence intervals (CI) was calculated, and sensitivity and specificity values were obtained from the ROC coordinates at relevant cut-off points.

## 4. Results

A total of 324 patients with angiographically confirmed coronary CTO scheduled for CTO revascularization procedure were included in this retrospective analysis. Of these, 208 patients (64.2%) were categorized into the poor collateral group (Rentrop grades 0–1), whereas 116 patients (35.8%) were classified into the good collateral group (Rentrop grades 2–3). Baseline demographic, clinical, laboratory, and echocardiographic parameters of the study population are shown in Table 1. The mean age did not differ significantly between the groups (63.51 ± 10.39 years in the poor collateral group vs. 62.26 ± 8.8 years in the good collateral group; *p* = 0.275). Likewise, the proportion of male patients was nearly equivalent (84.6% vs. 83.6%; *p* = 0.814). DM (38.5% vs. 41.4%, *p* = 0.482), hypertension (61.5% vs. 69.8%, *p* = 0.147), and current smoking (49.5% vs. 53.4%, *p* = 0.767) statuses were similar among groups. Body mass index (BMI) was modestly but significantly higher in patients with good collaterals (27.58 (26.14–30.01) vs. 26.92 (24.95–29.36) kg/m^2^; *p* = 0.040). White blood cell (WBC) counts were slightly elevated in the good collateral group (8.50 (7.21–9.98) vs. 8.29 (6.90–10.25) ×10^3^/µL; *p* = 0.076), although the difference was not statistically significant. Hemoglobin levels showed a trend toward being higher in the good collateral group (13.92 ± 1.89 g/dL vs. 13.51 ± 1.96 g/dL; *p* = 0.070). Renal function, assessed by estimated glomerular filtration rate (eGFR), was preserved in both groups but slightly lower in patients with good collaterals (84.66 (69.07–98.33) vs. 89.09 (70.51–100.2) mL/min/1.73 m^2^; *p* = 0.310). C-reactive protein (CRP) levels, a systemic marker of inflammation, did not differ significantly between groups (4.12 (3–6.95) vs. 4.47 (3–7)mg/L; *p* = 0.792). TC and LDL cholesterol (LDL-C) levels were similar between groups (*p* = 0.384 and *p* = 0.970, respectively). However, patients with good collaterals had significantly higher HDL cholesterol (HDL-C) (38 (33–45) vs. 36 (32–42) mg/dL; *p* = 0.042) and higher triglyceride levels (165 (116.25–228.5) vs. 147.5 (105–197.5) mg/dL; *p* = 0.040), (Table 1).

**Figure 1:**
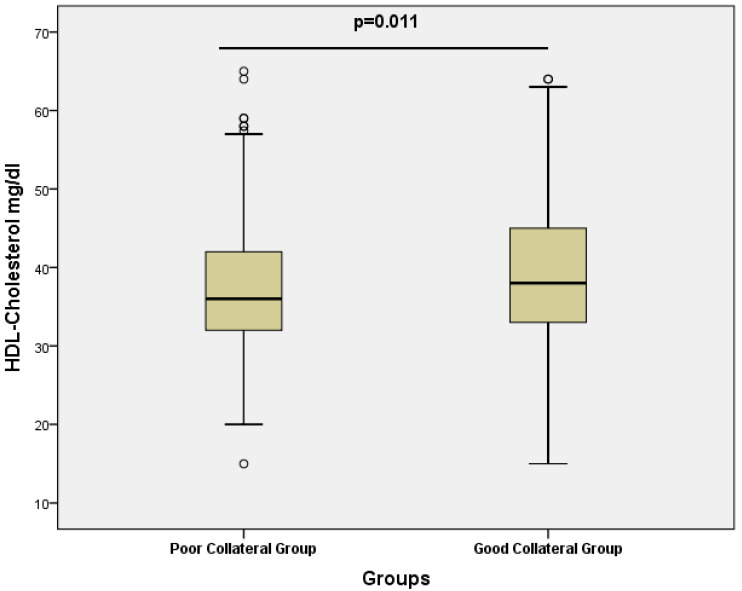
Box Plot graph depicting HDL Cholesterol levels (mg/dL) in the poor and good collateral groups.

**Figure 2:**
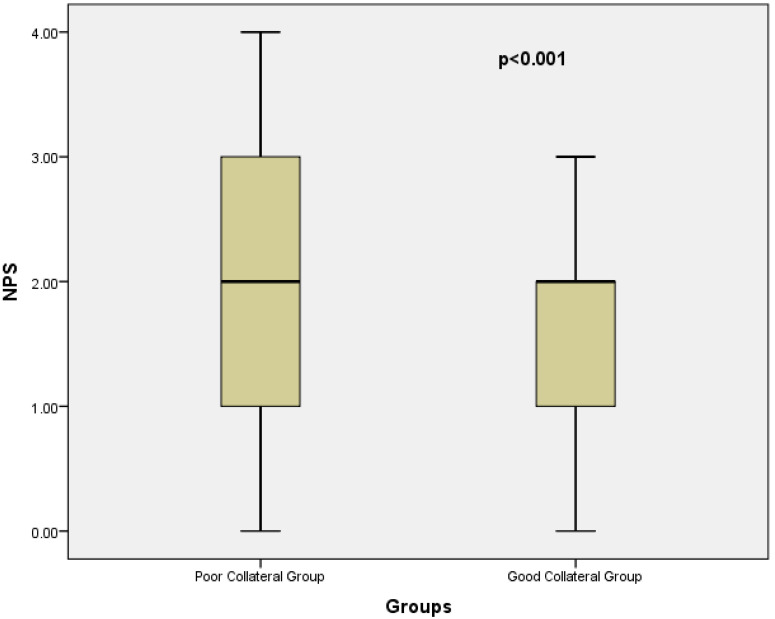
Box Plot graph depicting NPS (range 0–4) in the poor and good collateral groups.

The NPS demonstrated a highly significant difference between groups. Patients with poor collateral circulation had significantly higher NPS values compared to those with good collateral circulation (2 (2–3) vs. 2 (1–2); median (interquartile range, 25–75), *p* < 0.001). This finding highlights the strong association between worse inflammatory/nutritional status and impaired collateral vessel development. Multicollinearity among predictor variables was evaluated using VIF. All VIF values were well below the conventional threshold of 2, indicating that collinearity did not materially influence the regression analyses. Specifically, the VIF values were 1.019 for BMI, 1.063 for Hb, 1.051 for WBC, 1.077 for HDL-C, 1.078 for triglycerides, 1.038 for smoking, and 1.037 for hypertension. These findings confirmed the independence of the included variables in the regression models. To identify independent predictors of good collateral development, univariate and multiple binary logistic regression analyses were performed (Table 2). From the baseline characteristics, BMI, Hb, WBC, HDL-C, Triglycerides, NPS, smoking and hypertension met the prespecified threshold of *p* < 0.20 and were therefore tested in univariate logistic regression. Among these, only HDL-C and NPS remained statistically significant (*p* < 0.05) and were subsequently included in the multivariate model. Variables such as BMI and hemoglobin, despite showing *p*-values < 0.20 at baseline, were not retained as they did not achieve statistical significance in the univariate analysis. In the univariate binary logistic regression analysis, HDL-C (OR 1.029; 95% CI 1.003–1.055; *p* = 0.027) and NPS (OR 0.239; 95% CI 0.138–0.412; *p* < 0.001) were significantly associated with collateral status. Variables such as BMI, Hb, WBC, triglycerides, smoking, and hypertension did not reach statistical significance. Although triglyceride levels were significantly higher in the good collateral group at baseline (*p* = 0.040), they were not independently associated with collateral circulation in univariate logistic regression (*p* = 0.478) and therefore were not included in the multivariate model. In multiple binary logistic regression analysis, both HDL-C (OR 1.035; 95% CI 1.008–1.063; *p* = 0.011) and NPS (OR 0.226; 95% CI 0.130–0.393; *p* < 0.001) remained independent predictors of good collateral circulation. Box plot graphs depicting NPS and HDL-C levels in the poor, and good coronary collateral groups, including p values, are shown in Figure 1 and Figure 2. These findings highlight that higher HDL levels and a lower NPS are strongly and independently associated with the presence of well-developed CCC in patients with CTO.

**Figure 3:**
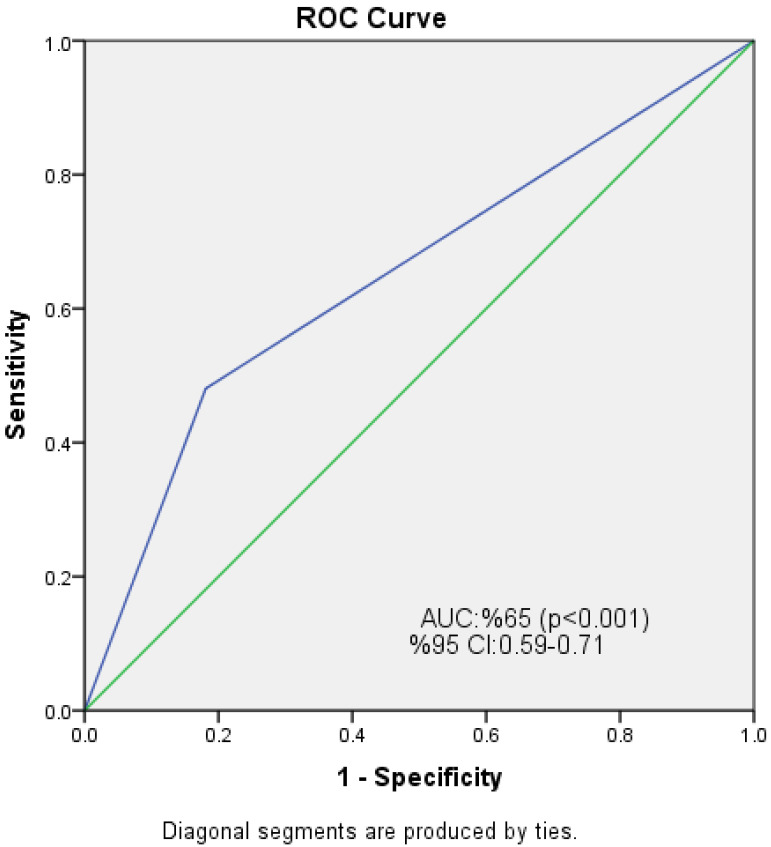
Receiver operating characteristic (ROC) curve of the Naples Prognostic Score (NPS) for predicting poor coronary collateral circulation. The area under the curve (AUC) was 0.65 (95% CI: 0.59–0.71, *p* < 0.001). At the cutoff of NPS ≥ 1.5, the sensitivity was 48.1% and the specificity was 81.9%.

The ROC analysis showed that NPS had a statistically significant ability to differentiate between poor and good collateral circulation. The AUC for NPS was 0.65 (95% CI: 0.59–0.71, *p* < 0.001). At a cutoff value of ≥1.5, NPS yielded a sensitivity of 48.1% and a specificity of 81.9% for predicting poor collateral development. Additionally, we present a new figure which consists of the ROC curve (Figure 3).

## 5. Discussion

In this retrospective study of 324 CTO patients undergoing CTO revascularization procedure, we demonstrated that NPS and HDL-C levels are independently associated with well-developed CCC. Specifically, a lower NPS (indicative of favorable inflammatory and nutritional status) and higher HDL-C levels were significantly associated with better coronary collateral formation, even after adjustment for other variables in binary logistic regression analysis. To the best of our knowledge this is the first study investigating the relationship between the coronary collateral degree and NPS.

The presence and adequacy of CCC are fundamental determinants of prognosis in patients with CAD, especially those with chronic total occlusion (CTO). Collateral vessels provide an alternative source of myocardial perfusion distal to an occlusion, thereby limiting ischemia, preserving left ventricular function, reducing infarct size, and lowering the risk of adverse outcomes, including mortality and ventricular arrhythmias [24]. A meta-analysis by Meier et al. showed that in CTO patients, those with good CCC had a 36% lower risk of mortality than those with poor CCC [25]. Another important point is that CTO revascularization procedures are increasing worldwide exponentially. Pre-procedural evaluation of the coronary collaterals between epicardial coronary arteries is indispensable [22]. Although a correlation with lower rates of acute myocardial infarction (AMI) and mortality has not been demonstrated, a meta-analysis conducted by Allahwala et al. has shown that well-developed coronary collaterals predict the success of CTO revascularization procedures [26]. Despite their clinical importance, there is striking inter-individual variability in collateral vessel growth, even among patients with similar anatomical burden of disease. This variability underscores the need to identify systemic and biological factors influencing collateral development.

NPS was first described by Galizia et al. in 2017, and its relationship with prognosis in colorectal cancer patients was investigated [11]. It was initially investigated in various cancer types and has subsequently gained increasing interest in recent years, with numerous studies focusing on its relationship with cardiovascular diseases [13,14,27]. The NPS integrates inflammatory and nutritional indices, including the NLR, the LMR, serum albumin, and TC. In this way, the NPS serves as a composite biomarker of systemic immune–nutritional status. Chronic systemic inflammation impairs endothelial progenitor cell mobilization, disrupts nitric oxide-mediated vasodilation, and inhibits angiogenic signaling pathways. These processes are essential for arteriogenesis [28,29]. Akkaya et al. investigated the role of Adropin, which has positive effects on the endothelium through NO and VEGF, on the CCC and they showed that adropin is associated with the well-developed CCC [29]. Mao et al. showed that systemic immune–inflammation index is, which represent the inflammatory status, associated with poor coronary collaterals [9]. Additionally, hypoalbuminemia and hypocholesterolemia reflect malnutrition and reduced substrate availability for endothelial repair, further attenuating vascular remodeling. Esenboğa et al. demonstrated that the Prognostic Nutritional Index (another marker of immune–nutritional status) was positively correlated with collateral development in CTO patients [30]. Together, these findings underscore the pivotal role of systemic metabolic and inflammatory homeostasis in shaping the coronary collateral response. NPS offers unique features with ability to reflect both inflammatory and nutritional status simultaneously.

In recent years, NPS has also been well studied in patients with cardiovascular disease. Saygı et al. showed that NPS is independently associated with the in-hospital mortality in patients with ST-Elevation myocardial infarction [17]. Gitmez et al. demonstrated that NPS independently predicted one-year mortality in patients with Non-ST elevation myocardial infarction [27]. Chen et al. showed that NPS is strongly associated with all-cause and cardiovascular mortality in patients with type 2 DM [31]. At the same time, Aydın et al. showed that NPS is an independent predictor of long-term mortality in patients with heart failure in a large-scale study [32]. A recent study by Dündar et al. offers compelling evidence that NPS is independently associated with long-term mortality in patient with CTO undergoing percutaneous coronary intervention [33].

Our results also indicated that HDL-C levels are independently associated with good coronary collateral development. The association between HDL-C and collateral formation aligns with its established vasoprotective functions, including reverse cholesterol transport, antioxidation, anti-inflammation, and endothelial repair, which are all critical to vascular remodeling [24,34]. Furthermore, Lee et al. found that HDL functionality, as measured by cholesterol efflux capacity (CEC), was more strongly associated with collateral grade than HDL levels themselves [34]. Notably, CEC emerged as an independent positive predictor of CCC in patients with CTO. A broader meta-analysis further confirmed that elevated CEC is associated with lower incidence of adverse cardiovascular events [35]. Unfortunately, we were unable to take any measurements related to CEC. Emerging studies highlight additional lipid–inflammatory indices impacting collateral formation. For example, the atherogenic index of plasma (AIP), calculated as log (TG/HDL-C), was found to be independently associated with poor CCC in a CTO cohort [36]. As with all previous findings, our results indicated that HDL-C is an independent predictor of good CCC.

Identifying NPS and HDL-C (particularly functional HDL, as measured by CEC) as predictors of collateral formation has significant clinical value. Since both are derived from routine laboratory measures, they are accessible tools for stratifying CTO patients by collateral potential. Patients with elevated NPS and low HDL may require more intensive management, including nutritional optimization and anti-inflammatory strategies. Furthermore, interventions that improve HDL functionality, such as lifestyle modifications or emerging therapies, and interventions-medications that improve inflammatory status could selectively enhance collateral growth; however, prospective validation is needed.

### 5.1. Future Perspectives

Future research should go beyond validating our findings and explore strategies for improving collateral vessel development in CTO. Future studies that may fill the gaps of this topic in the literature may be as follows:

- Prospective, multicenter studies are necessary to incorporate systemic biomarkers, such as the NPS and HDL-C, into comprehensive risk prediction models alongside angiographic and clinical variables.
- Mechanistic studies examining the influence of inflammation, nutrition, and lipid metabolism on arteriogenesis could clarify causal pathways. Therapeutic interventions that target these mechanisms also merit investigation.
- For instance, structured exercise programs and anti-inflammatory therapies could enhance collateral growth by improving endothelial function, and dietary and metabolic strategies could optimize nutritional and lipid profiles. Additionally, emerging approaches focused on HDL functionality, such as interventions designed to increase cholesterol efflux capacity, should be evaluated for their ability to promote vascular remodeling.
- Finally, the integration of advanced imaging and hemodynamic techniques, including quantitative perfusion imaging and invasive indices of collateral flow, could enable a more precise assessment of collateral function and response to therapy. These research directions show promise in translating biomarker-based risk assessment into personalized therapeutic strategies for CTO patients.

### 5.2. Limitations

This study has several limitations. First, the retrospective, single-center design limits generalizability, introduces potential selection bias, and cannot exclude residual confounding. Because most patients were referred for revascularization procedures due to its feature of being a reference center, referral bias may also have influenced the study population. Second, CCC was assessed using the Rentrop angiographic classification, which is a semi-quantitative, observer-dependent tool that reflects angiographic visibility rather than functional capacity. More accurate methods, such as the collateral flow index, would provide greater precision. Future studies should therefore incorporate functional measures, such as collateral flow index or myocardial perfusion imaging, to better characterize collateral capacity. Third, NPS is a useful composite index, but it is influenced by dynamic laboratory parameters, so a single measurement may not fully capture chronic inflammatory-nutritional status. On the other hand it should be carefully noted that the cut-off values for the components of the NPS were adopted from the original oncology literature and subsequently applied in cardiovascular studies without recalibration. While this approach facilitates comparability across studies, disease-specific thresholds have not yet been validated in the CTO population. Prospective investigations are needed to establish optimal cutoffs for NPS components in this clinical setting, which may enhance its predictive accuracy. Fourth, although HDL-C was identified as an independent predictor, we did not assess HDL functionality, particularly cholesterol efflux capacity. This is a more reliable indicator of cardiovascular outcomes and collateral development. It should be acknowledged that the ROC analysis yielded an AUC of 0.65, which falls within the range typically interpreted as indicating modest discriminative accuracy. This underscores that NPS alone may not serve as a strong standalone predictor of coronary collateral circulation. However, despite this limitation, the association between NPS and CCC remained statistically significant, and HDL-C also emerged as an independent predictor. These findings suggest that while the predictive power of NPS is modest, its clinical relevance lies in highlighting the contribution of inflammatory and nutritional status to collateral vessel development.

Finally, the absence of longitudinal outcome data prevents us from determining the impact of the NPS and HDL on clinical outcomes. This limits the ability to assess the prognostic implications of these biomarkers, and prospective follow-up studies are needed to clarify their value in predicting long-term outcomes. Nevertheless, the originality of this work lies in being the first study to investigate the association of NPS with coronary collateral circulation in CTO patients. Prior studies in coronary artery disease have largely focused on single inflammatory markers such as CRP, NLR, or PLR, or on nutritional indices like the Prognostic Nutritional Index (PNI) and the CONUT score. By contrast, the NPS is an integrative tool that simultaneously incorporates both inflammatory and nutritional parameters, offering a more comprehensive reflection of the systemic milieu that influences vascular remodeling. Our study extends the application of NPS—originally validated in oncology and more recently explored in general CAD cohorts—into the specific context of CTO, where collateral vessel development has profound clinical implications. Furthermore, by demonstrating that HDL-C, a lipid marker with known vascular protective properties, and NPS, an immune–nutritional composite index, are independent predictors of collateral formation, our work uniquely bridges the domains of lipid metabolism, systemic inflammation, and nutrition in relation to coronary collateral growth. This combined approach provides novel insights into the systemic determinants of collateral vessel development and positions our study as an original contribution to the literature on risk stratification in CTO.

## 6. Conclusions

In patients with CTO, the development of CCC is a crucial determinant of myocardial protection and long-term prognosis. In this study, we demonstrated that both the NPS and HDL-C independently predict the degree of collateral vessel formation. Specifically, higher NPS values reflecting adverse inflammatory and nutritional status were associated with poor collateral development, while elevated HDL-C levels correlated with the presence of well-developed collaterals. These findings underscore the pivotal interplay between systemic immune–nutritional balance, lipid metabolism, and vascular adaptation in the pathophysiology of collateral growth. Given the affordability and accessibility of both NPS and HDL-C as clinical measures, their incorporation into risk stratification protocols could serve as a pragmatic approach to identifying patients who are at risk of inadequate collateralization. Such patients may benefit from intensified preventive strategies and closer clinical monitoring.

Future research should validate these results in larger, prospective, multicenter cohorts using functional collateral assessment. Additionally, research should investigate whether targeted interventions such as optimizing nutrition, reducing systemic inflammation, and enhancing HDL functionality can improve collateral vessel development and clinical outcomes in CTO.

## Figures and Tables

**Table 1:** Baseline demographic, clinical, laboratory and echocardiographic parameters of the study population.

Variable	Poor Collateral (n = 208, 64.2%)	Good Collateral (n = 116, 35.8%)	*p*-Value
Age (years)	63.51 ± 10.39	62.26 ± 8.8	0.275
Gender (male, %)	84.6%	83.6%	0.814
BMI (kg/m^2^)	26.92 (24.95–29.36)	27.58 (26.14–30.01)	0.04 **
WBC (10^3^/µL)	8.29 (6.90–10.25)	8.50 (7.21–9.98)	0.076
Hb (g/dL)	13.51 ± 1.96	13.92 ± 1.89	0.07
GFR (mL/min/1.73m^2^)	89.09 (70.51–100.2)	84.66 (69.07–98.33)	0.31
Total Cholesterol (mg/dL)	152.5 (130–182)	160.1 (131–204.53)	0.384
LDL (mg/dL)	84.30 (64–106.75)	82.6 (64.1–119.86)	0.970
HDL (mg/dL)	36 (32–42)	38 (33–45)	0.042 **
Triglycerides (mg/dL)	147.5 (105–197.5)	165 (116.25–228.5)	0.040 **
CRP (mg/L)	4.47 (3–7)	4.12 (3–6.95)	0.792
Systolic BP (mmHg)	110 (100–120)	110 (110–120)	0.364
Diastolic BP (mmHg)	70 (65–80)	70 (70–75)	0.741
NPS	2 (2–3)	2 (1–2)	<0.001 **
Statin use (%)	84.6%	85.3%	0.861
LVEF (%)	58 (53–62)	59 (52–64)	0.883
Diabetes mellitus (%), n	38.5% (80)	41.4% (48)	0.482
Hypertension (%), n	61.5% (128)	69.8% (81)	0.147
Smoking (%), n	49.5% (103)	53.4% (62)	0.767
ACE inhibitor-ARB use (%), n	56.7% (118)	61.2% (71)	0.481
Beta-blocker use (%), n	72.6% (151)	72.4% (84)	1.000

**Acronyms:** All continuous variables are presented with appropriate precision (mean ± SD for normally distributed variables; median with interquartile range for skewed variables). Values are presented as mean ± standard deviation, median (interquartile range, 25–75), or n (%). BMI = Body Mass Index; WBC = White Blood Cell count; Hb = Hemoglobin; GFR = Glomerular Filtration Rate; LDL = Low-Density Lipoprotein; HDL = High-Density Lipoprotein; CRP = C-reactive protein; BP = Blood Pressure; NPS = Naples Prognostic Score; LVEF = Left Ventricular Ejection Fraction; ACE = Angiotensin-Converting Enzyme; ARB = Angiotensin Receptor Blocker. ** denotes statistical significance.

**Table 2:** Binary logistic regression analyses to determine the independent associates of the well-developed coronary collateral circulation.

Variables	Univariate OR (95% CI)	*p*	Multiple OR (95% CI)	*p*
BMI (kg/m^2^)	1.050 (0.991–1.112)	0.098	-	-
Hb (g/dL)	1.116 (0.991–1.257)	0.071	-	-
WBC (10^3^/µL)	0.989 (0.898–1.088)	0.818	-	-
HDL cholesterol (mg/dL)	1.029 (1.003–1.055)	0.027*	1.035 (1.008–1.063)	0.011 *
Triglyceride (mg/dL)	1.001 (0.998–1.003)	0.478	-	-
NPS	0.239 (0.138–0.412)	<0.001*	0.226 (0.130–0.393)	<0.001 *
Smoking	0.916 (0.731–1.149)	0.449	-	-
Hypertension	0.691 (0.426–1.123)	0.136	-	-

**Acronyms:** Hb, Hemoglobin; HDL, high-density lipoprotein; WBC, white blood cell; NPS, Naples prognostic score; BMI, body mass index. * a *p* value < 0.05 denotes statistical significance.

## Data Availability

The data presented in this study are available on request from the corresponding author.
